# Drug Markets at Home and Abroad

**Published:** 1913-11-15

**Authors:** 


### Drug Markets at Home and Abroad.

At an evening meeting of the Pharmaceutical Society held at 17 Bloomsbury Square, on November 11, Professor H. G. Greenish, Dean of the School of Pharmacy, exhibited and described a number of lantern-slides illustrating the Drug Markets at Home and Abroad. London is by far the most important Drug market in the world, but a large business in drugs is also done at other centres, notably at Hamburg and Amsterdam, the latter place being now the market for cinchona bark. The lantern-slides illustrated among other things the curious methods in which drugs are packed by native collectors for export. For instance, aloes from the West Indies usually arrives in wooden cases in which bottled spirits have been exported, while aloes from Zanzibar comes over in goat-skins. Cassia comes from Java in baskets made of plaited split cane, and cloves are sent from Zanzibar in mats made from interlaced strips of cocoanut-leaves.

Professor Greenish referred to the practice of adulteration which is prevalent among native collectors, and explained that the raw drugs purchased by wholesale dealers have to be submitted to the process of hand-picking before they are passed on to the chemist, or, of course, to the hospital pharmacist. Opium is sometimes loaded with lead shot; buchu leaves sometimes contain as much as 25 per cent. of stalks, and packages of senna often contain sand. All this extraneous matter has to be removed from the drugs before they are ready for pharmaceutical uses.

